# The reliability of a quality appraisal tool for studies of diagnostic reliability (QAREL)

**DOI:** 10.1186/1471-2288-13-111

**Published:** 2013-09-09

**Authors:** Nicholas Lucas, Petra Macaskill, Les Irwig, Robert Moran, Luke Rickards, Robin Turner, Nikolai Bogduk

**Affiliations:** 1 Screening and Test Evaluation Program, Sydney School of Public Health, University of Sydney, Sydney, Australia; 2 School of Health Science, UNITEC, Auckland, New Zealand; 3 Private Practice, Sydney, Australia; 4 Department of Clinical Research, Newcastle Bone and Joint Institute, Royal Newcastle Centre, University of Newcastle, Newcastle, Australia

**Keywords:** Reliability, Quality appraisal, Systematic review, Evidence-based medicine

## Abstract

**Background:**

The aim of this project was to investigate the reliability of a new 11-item quality appraisal tool for studies of diagnostic reliability (QAREL). The tool was tested on studies reporting the reliability of any physical examination procedure. The reliability of physical examination is a challenging area to study given the complex testing procedures, the range of tests, and lack of procedural standardisation.

**Methods:**

Three reviewers used QAREL to independently rate 29 articles, comprising 30 studies, published during 2007. The articles were identified from a search of relevant databases using the following string: “Reproducibility of results (MeSH) OR reliability (t.w.) AND Physical examination (MeSH) OR physical examination (t.w.).” A total of 415 articles were retrieved and screened for inclusion. The reviewers undertook an independent trial assessment prior to data collection, followed by a general discussion about how to score each item. At no time did the reviewers discuss individual papers. Reliability was assessed for each item using multi-rater kappa (κ).

**Results:**

Multi-rater reliability estimates ranged from κ = 0.27 to 0.92 across all items. Six items were recorded with good reliability (κ > 0.60), three with moderate reliability (κ = 0.41 - 0.60), and two with fair reliability (κ = 0.21 - 0.40). Raters found it difficult to agree about the spectrum of patients included in a study (Item 1) and the correct application and interpretation of the test (Item 10).

**Conclusions:**

In this study, we found that QAREL was a reliable assessment tool for studies of diagnostic reliability when raters agreed upon criteria for the interpretation of each item. Nine out of 11 items had good or moderate reliability, and two items achieved fair reliability. The heterogeneity in the tests included in this study may have resulted in an underestimation of the reliability of these two items. We discuss these and other factors that could affect our results and make recommendations for the use of QAREL.

## Background

The Quality Appraisal for Reliability Studies (QAREL) checklist is an appraisal tool recently developed to assess the quality of studies of diagnostic reliability [1]. When QAREL was first accepted for publication in 2009, no other quality appraisal tool was widely accepted for use in systematic reviews of reliability studies, and QAREL was therefore developed to fill this gap. Since then, both the COSMIN [2] and GRRAS [3] checklists have been published. COSMIN, deals with the methodological quality of agreement and reliability studies, whereas GRRAS deals with the reporting of such studies. This paper focuses specifically on the evaluation of the reliability of QAREL.

QAREL is an 11-item checklist that covers 7 key domains, those being the spectrum of subjects; the spectrum of examiners; examiner blinding; the order effects of examination; the suitability of the time-interval between repeated measurements; appropriate test application and interpretation; and appropriate statistical analysis. Using this checklist, reviewers are able to evaluate individual studies of diagnostic reliability in the preparation of systematic reviews.

QAREL was developed in consultation with a reference group of individuals with expertise in diagnostic research and quality appraisal [1]. This panel identified specific areas of bias and error in reliability studies to derive relevant items for potential inclusion on a new quality appraisal tool. Systematic reviews of reliability studies were also examined to identify existing quality appraisal tools [4-10]. In addition, the STARD [11] and QUADAS [12] resources were reviewed for additional items not already identified. Using an iterative process, members of the panel reviewed the proposed items and reduced the list to those considered essential for assessing study quality.

We also developed an instruction document and data extraction form for use in systematic reviews [1]. The data extraction form is to be used in conjunction with QAREL to help systematic reviewers extract relevant information from primary studies.

It is necessary to evaluate the reliability of QAREL, where reliability is a measure of the chance corrected agreement between different reviewers who independently rate the same set of papers. The aim of this study was to investigate the inter-rater reliability of each item on the QAREL checklist. The reliability of physical examination was chosen as the topic area for this study as there is high variability in the performance, interpretation and reporting of physical examination procedures, and this provided a challenging context in which to evaluate the reliability of QAREL.

## Methods

Three reviewers (NL, RM, LR) participated in this study designed to evaluate the inter-rater reliability of each item on QAREL. The University of Sydney Human Research Ethics committee granted approval for the study.

All reviewers were qualified health professionals and had experience in physical examination procedures. Each had experience in the critical appraisal of research papers, and had participated in formally reviewing papers for systematic reviews. Two reviewers (NL, RM) were involved in the development of QAREL.

A search of MEDLINE, CINAHL, AMED and SCOPUS was conducted to locate papers on the reliability of physical examination published from January 2007 through December 2007. The search string used to locate potential papers was “Reproducibility of results (MeSH) OR reliability (t.w.) AND Physical examination (MeSH) OR physical examination (t.w.). No limits were placed on the source title for the published paper, nor on the type of physical examination procedure reported.

A total of 415 records were retrieved and screened for potential inclusion in the study. Only articles that reported on the reliability of physical examination procedures were included. A total of 29 articles, comprising 30 studies, were retrieved and included in this study [13-40].

The reviewers received basic written instructions regarding the use of QAREL [1]. Each item on the checklist can be rated as ‘Yes’, ‘No’, or ‘Unclear’, and certain items can be rated as ‘Not Applicable’. Reviewers independently performed a trial assessment of each paper, followed by a meeting with members of the reference group involved in the development of QAREL to establish baseline criteria for the interpretation of each item. At no time did the reviewers discuss individual studies, which ensured that each reviewer remained blinded to the opinions and findings of other reviewers for each study. Reviewers discussed the general interpretation of individual items on QAREL and outlined general areas of ambiguity for certain items.

Following the meeting between reviewers and the reference group, each reviewer independently rated each paper. Reviewers were not permitted to communicate about the checklist or about the individual papers being reviewed. Completed data collection forms were returned for reliability (*κ*) analysis.

### Analysis

Data were analysed for reliability using *kappa* (*κ*) for multiple raters [41]. Each response option was recorded as a category, including ‘unclear’ and ‘not applicable’. All computations were performed using STATA 8.2 (StataCorp TX, USA) *Kappa* is a chance corrected measure of inter-rater reliability, and ranges from −1 to +1, with +1 being perfect agreement, –1 being perfect disagreement, and zero being agreement no better than chance. In this study, kappa was interpreted as unreliable (κ < 0.00), poor (κ = 0.01 – 0.20), fair (κ = 0.21 – 0.40), moderate (κ = 0.41 – 0.60), good (κ = 0.61 – 0.80) and very good (κ = 0.81 – 1.00). A 95% confidence interval for kappa was computed using the test-based standard error. For this study, reliability was considered acceptable if it was moderate or higher.

## Results

The estimates of multi-rater reliability for each item are presented in Table 1. The multi-rater scores for individual items ranged from κ 0.27 to κ 0.92, with one item reaching very good reliability (Item 3), eight achieving good or moderate reliability (Items 2, 4 – 9, 11), and two reaching fair reliability (Items 1, 10).

**Table 1:** Multi-rater reliability for reviewers rating of 30 studies of diagnostic reliability using QAREL

**Item**	**Item description (abbreviated)**	**Subsequent evaluation**	
		**κ**	**95% CI**
1	Was the sample of subjects representative?	0.27	(0.11, 0.42)
2	Was the sample of raters representative?	0.59	(0.43, 0.74)
3	Were raters blinded to the findings of other raters?	0.92	(0.76, 1.00)
4	Were raters blinded to their own prior findings?	0.78	(0.62, 0.94)
5	Were raters blinded to the accepted reference standard?	0.66	(0.49, 0.82)
6	Were raters blinded to clinical information not part of test	0.51	(0.37, 0.64)
7	Were raters blinded to additional non-clinical cues?	0.59	(0.39, 0.78)
8	Was the order of examination varied?	0.71	(0.58, 0.84)
9	Was the time interval between repeated measures appropriate?	0.69	(0.50, 0.88)
10	Was the test applied correctly and interpreted appropriately?	0.35	(0.18, 0.51)
11	Were appropriate statistical measures of agreement used?	0.73	(0.54, 0.92)

κ = multi-rater kappa. 95% CI = 95% confidence interval.

### Reliability of each item

Item 1, regarding the representativeness of subjects, was reported with fair reliability (κ =0.27). The reviewers identified “subject representativeness” as a difficult item to rate because each paper in this study presented a different diagnostic test procedure. Under normal circumstances, the scope of a systematic review would limit the number of tests making it possible for reviewers to identify and agree upon appropriate criteria thereby making judgments for this item more straightforward. In this evaluation 10 studies were classified as “Yes” and three studies were classified as “No” by all 3 raters. Two raters agreed on “yes” for 12 studies, “No” for 3 studies and “Unclear” for 1 study.

Reviewers also expressed difficulty rating Item 2, regarding the representativeness of the raters. This item, however, achieved moderate reliability (κ = 0.59). All three raters agreed on “Yes” for 15 studies, “No” for 2 studies and “unclear” for 4 studies. Two raters agreed on “yes” for 5 studies, “No” for 1 study, and “Unclear” for 2 studies.

For Item 3, reviewers reliably reported whether the raters in a given study were blinded to the findings of other raters. This item, which only has relevance to studies of inter-rater reliability, was reported with very good (κ= 0.92) reliability. All three reviewers selected “Yes” for 18 studies, “Unclear” for 5 studies and “Not Applicable” for 5 studies. “No” was not recorded for any study.

The purpose of item 4 is to identify if raters had any prior knowledge of the test outcome for a particular subject before rating them in the study. There are two possible situations in which this might occur. First, in studies of intra-rater reliability, the rater may recall their findings from the first ‘rating’ when they rate the subject a second time. The second possibility is that the rater may have performed the test on a subject prior to their enrolment in the study. For example, subjects may have been recruited from the rater’s own list of patients, and the rater may recall examination findings from their prior assessment of the patient. This item achieved good reliability (κ = 0.78). All three reviewers selected “Not Applicable” for 20 studies, “Yes” for 5 studies and “Unclear” for one study. “No” was not recorded for any study.

Item 5 concerns the blinding of raters to the results of the accepted reference standard. This item achieved good reliability (κ =0.66). All three reviewers selected “Not Applicable” for 22 studies, “Yes” for 2 studies and “Unclear” for one study. “No” was not recorded for any study.

Item 6 refers to whether raters were blinded to clinical information that was not intended to form part of the test procedure. This item was found to be moderately reliable (κ=0.51). All three raters agreed on “Yes” for five studies and “Unclear” for 13 studies. The remaining responses were spread across all categories.

The purpose of item 7 is to identify if raters had access to non-clinical information that was not intended to form part of the test procedure. Reliability may be influenced by the recognition of additional cues such as tattoos, scars, voice accent and unique identifying features on imaging films. The reviewers discussed that they could think of a large number of potential ‘additional cues’ that might be important for each study, and found it difficult to judge this item without predetermined criteria. Reliability for this item was moderate (κ = 0.59). All three reviewers classified 22 studies as “Unclear” for this item and three studies as “Yes”. Only a single reviewer selected “No” for a single study.

Item 8 requires reviewers to consider the order of examination and if it was varied during the study. This item was reported with good reliability (κ = 0.71). All three raters agreed on “Yes” for 10 studies, “No” for one study, “Unclear” for 7 studies and “Not Applicable” for 3 studies.

Item 9 considers the time interval between repeated test applications. This item achieved good reliability (κ = 0.69). All three raters agreed on “Yes” for 24 studies and “Unclear” for 3 studies. Only a single reviewer selected “No” for a single study.

Item 10 requires reviewers to consider if the test has been applied correctly and interpreted appropriately. This item was reported with fair reliability (κ=0.35). Interpretation of these results should take into account that each study reported a different physical examination test. Under more typical systematic review conditions, only one or a small number of related tests would be reported. All 3 reviewers selected “Yes” for 23 studies and “No” for one study. A single reviewer selected “Unclear” for 4 studies, “Yes” for one study and “No” for one study.

Item 11 requires reviewers to consider if the statistical analysis used was appropriate. Reliability for this item was found to be good (κ = 0.73). All three reviewers agreed on “Yes” for 26 studies and “No” for 2 studies.

## Discussion

In this study we evaluated the reliability of individual items on the QAREL checklist in the area of physical examination. We found that the majority of items were reported with either moderate or good reliability, with two items achieving fair reliability. From these results, we consider that QAREL is a reliable tool for the assessment of studies of diagnostic reliability, and we emphasize that reviewers should have the opportunity to discuss the criteria by which to rate individual studies, as is typical in the preparation of systematic reviews. We also recommend further studies to evaluate the reliability of QAREL as used by different examiners and in different contexts.

As mentioned in the background, COSMIN is a related tool and has also been published and assessed for reliability [42]. COSMIN was developed to evaluate the measurement properties of health measurement instruments, of which reliability is one property, whereas QAREL was developed to specifically evaluate reliability.

COSMIN has been evaluated for inter-rater reliability [42] in a study comprising 88 examiners who used COSMIN to rate a total of 75 papers. Of the 14 COSMIN reliability items, good reliability (κ = 0.72) was achieved for one item, and moderate reliability (κ = 0.41-0.60) was achieved for 5 items. For the reliability of items on QAREL, 6 of 11 items had good reliability, and 3 had moderate reliability. The QAREL and COSMIN reliability studies differ markedly in their design, however, which makes it difficult to compare reliability between the items or constructs that they have in common.

Four main factors should be taken into consideration in the interpretation of the results. First, reliability of physical examination is a challenging area to investigate. Physical examination procedures are subject to variability in both test application and interpretation. In addition, many of the disorders that are evaluated by physical examination procedures do not have an accepted reference standard by which to confirm test results. This absence makes it difficult for reviewers to determine if any differences observed in repeated test outcomes are attributable to real changes in the underlying disorder, or variability in the test application and interpretation. For example, Item 9 is concerned with whether the time interval between repeated applications of the same test was appropriate, yet this knowledge can only be determined by application of an accepted reference standard. This example highlights the need for reviewers to agree upon criteria for rating this item prior to undertaking reviews of individual studies.

Second, this study is atypical because each of the articles reports the reliability of a different physical examination procedure, with no two articles reporting on the same test. This introduced an unusually high level of variability in this study in terms of the test procedures, type of patients or subjects, type of examiners, and types of disorder. Under normal conditions, QAREL would more likely be used to evaluate a group of related papers, each reporting the reliability of the same test in different patients groups and as performed by different examiners. In that context, reviewers would establish agreed criteria by which to rate each item on QAREL, prior to evaluating the papers. This study, therefore, evaluated QAREL under challenging circumstances, and this may have led to lower reliability estimates.

A third factor that should be mentioned is that the estimated reliability (kappa) for each item is affected by the distribution of responses across the available categories for that item. A large imbalance in the number of responses across categories, as occurred for item 10, can result in a low estimate for reliability (kappa) even when observed agreement between raters is high.

Lastly, this study comprised three reviewers and 29 papers reporting studies of reliability in the area of physical medicine. Further evaluation is warranted to assess the reliability of QAREL in other contexts, and the effect of training. A larger study would provide scope to investigate the effect of reviewer experience and training.

## Conclusion

In this study, we found that QAREL was a reliable assessment tool for studies of diagnostic reliability when reviewers had the opportunity to discuss the criteria by which to interpret each item. Reliability for 9 out of 11 items was moderate or good, and fair for 2 (items 1 and 10). The results for these two items were likely affected by the heterogeneous group of papers evaluated in this study and the challenges inherent in the field of physical examination. If reviewers utilize QAREL after agreement on the criteria by which they will make judgments for each item, they can expect the tool to be reliable. Further testing of the reliability of QAREL in different contexts is needed to further establish the reliability of this tool.

## Competing interests

The authors declare that they have no competing interests.

## Authors’ contributions

The authors of this paper are Nicholas Lucas (NL), Petra Macaskill (PM), Les Irwig (LI), Rob Moran (RM), Luke Rickards (LR), Robin Turner (RT), and Nikolai Bogduk (NB). The author contributions were: NL conceived of the study, designed the initial study protocol and implemented the study. PM, LI and NB provided advice on the study protocol and participated in the study as the reference group. NL, RM, an LR undertook the reliability study and rated all papers. NL wrote the first draft of the paper. All authors contributed to and approved the final version of the paper.

## Pre-publication history

The pre-publication history for this paper can be accessed here:

http://www.biomedcentral.com/1471-2288/13/111/prepub

## References

[B1] Lucas NP Macaskill PM Irwig L Bogduk N The development of a quality appraisal tool for studies of diagnostic reliability (QAREL) J Clin Epidemiol 2010 63 854 861 10.1016/j.jclinepi.2009.10.002 20056381

[B2] Mokkink LB Terwee CB Patrick DL Alonso J Stratford PW Knol DL Bouter LM de Vet HCW The COSMIN checklist for assessing the methodological quality of studies on measurement properties of health status measurement instruments: an international Delphi study Qual Life Res 2010 19 539 549 10.1007/s11136-010-9606-8 20169472 PMC2852520

[B3] Kottner J Audige L Brorson S Donner A Gajewski BJ Hrobjartsson A Guidelines for Reporting Reliability and Agreement Studies (GRRAS) were proposed J Clin Epidemiol 2011 64 96 106 10.1016/j.jclinepi.2010.03.002 21130355

[B4] Gemmell H Miller P Interexaminer reliability of multidimensional examination regimens used for detecting spinal manipulable lesions: a systematic review Clin Chiropr 2005 8 199 204 10.1016/j.clch.2005.09.002

[B5] Hestboek L Leboeuf-Yde C Are chiropractic tests for the lumbo-pelvic spine reliable and valid? A systematic critical literature review J Manipulative Physiol Ther 2000 23 258 275 10820299 10.1067/mmt.2000.106097

[B6] Hollerwoger D Methodological quality and outcomes of studies addressing manual cervical spine examinations: a review Man Ther 2006 11 93 98 10.1016/j.math.2005.12.001 16483829

[B7] May S Littlewook C Bishop A Reliability of procedures used in the physical examination of non-specific low back pain: a systematic review Aust J Physiother 2006 52 91 102 10.1016/S0004-9514(06)70044-7 16764546

[B8] Seffinger MA Najm WI Mishra SI Adams A Dickerson VM Murphy LS Reliability of spinal palpation for diagnosis of back and neck pain: a systematic review of the literature Spine 2004 29 E413 25 10.1097/01.brs.0000141178.98157.8e 15454722

[B9] Stochkendahl MJ Christensen HW Hartvigsen J Vach W Haas M Hestbaek L Manual examination of the spine: a systematic critical literature review of reproducibility J Manipulative Physiol Ther 2006 29 475 85 485 e1-10 10.1016/j.jmpt.2006.06.011 16904495

[B10] Van Trijffel E Anderegg Q Bossuyt PMM Lucas C Inter-examiner reliability of passive assessment of intervertebral motion in the cervical and lumbar spine: A systematic review Man Ther 2005 10 256 269 10.1016/j.math.2005.04.008 15994114

[B11] Bossuyt PM Reitsma JB Bruns DE Gatsonis CA Glasziou PP Irwig LM Towards complete and accurate reporting of studies of diagnostic accuracy: The STARD Initiative Ann Intern Med 2003 138 1 40 44 10.7326/0003-4819-138-1-200301070-00010 12513043

[B12] Whiting P Rutjes AW Reitsma JB Bossuyt PM Kleijnen J The development of QUADAS: a tool for the quality assessment of studies of diagnostic accuracy included in systematic reviews BMC Med Res Methodol 2003 3 25 10.1186/1471-2288-3-25 14606960 PMC305345

[B13] Bertilson B Grunnesjo M Johansson S-E Pain drawing in the assessment of neurogenic pain and dysfunction in the neck/shoulder region: Inter-examiner reliability and concordance with clinical examination Pain Med 2007 8 134 146 10.1111/j.1526-4637.2006.00145.x 17305685

[B14] Bremander AB Dahl LL Roos EM Validity and reliability of functional performace tests in meniscectomized patients with or without knee osteoarthritis Scand J Med Sci Sports 2007 17 120 127 17394472 10.1111/j.1600-0838.2006.00544.x

[B15] Brushoj C Langberg H Larsen K Reliability of normative values of the foot line test: a technique to assess foot posture J Ortho Sports Phys Ther 2007 37 703 707 10.2519/jospt.2007.2525 18069142

[B16] Bybee RF Dionne CP Interrater agreement on assessment, diagnosis, and treatment for neck pain by trained physical therapist students J Phys Ther Edu 2007 21 39 47

[B17] Cook C Massa L Harm-Emandes I Segneri R Adcock J Kennedy C Figuers C Interrater reliability and diagnostic accuracy of pelvic girdle pain classification J Manipulative Physiol Ther 2007 30 252 258 10.1016/j.jmpt.2007.03.008 17509433

[B18] De Jong LD Nieuwboer A Aufdemkampe G The hemiplegic arm: Interrater reliability and concurrent validity of passive range of motion measurements Disability Rehab 2007 29 1442 1448 10.1080/09638280601056145 17729091

[B19] Dionne C Bybee RF Tomaka J Correspondence of diagnosis to initial treatment for neck pain Physiotherapy 2007 93 62 68 10.1016/j.physio.2006.11.001

[B20] Gladman DD Inman RD Cook RJ van der Heijde D Landewe RMB International spondyloarthritis interobserver reliability exercise. The INSPIRE study: I. Assessment of spinal measures J Rheumatol 2007 34 1733 1739 17611985

[B21] Gladman DD Inman RD Cook RJ Maksymowych WP Braun J International spondyloarthritis interobserver reliability exercise. The INSPIRE study: II. Assessment of peripheral joints, enthesitis, and dactylitis J Rheumatol 2007 34 1740 1745 17659754

[B22] Hacker MR Funk SM Manco-Johnson MJ The Colorado haemophilia paediatric joint physical examination scale: Normal values and interrater reliability Haemophilia 2007 13 71 78 10.1111/j.1365-2516.2006.01387.x 17212728

[B23] Hickey BW Milosavljevic S Bell ML Milburn PD Accuracy and reliability of observational motion analysis in identifying shoulder symptoms Man Ther 2007 12 263 270 10.1016/j.math.2006.05.005 16973403

[B24] Hungerford BA Gilleard W Moran M Emmerson C Evaluation of the ability of physical therapists to palpate intrapelvic motion with the stork test on the support side Phys Ther 2007 87 879 887 10.2522/ptj.20060014 17472953

[B25] Kim Y-S Kim J-M Ha K-Y Choy S Joo M-W The passive compression test: A new clinical test for superior labral tears of the shoulder Am J Sports Med 2007 35 1489 1494 10.1177/0363546507301884 17478654

[B26] Kim HW Ko YJ Rhee WI Lee JS Lim JE Interexaminer reliability and accuracy of posterior superior iliac spine and iliac crest palpation for spinal level estimations J Manipulative Physiol Ther 2007 30 386 389 10.1016/j.jmpt.2007.04.005 17574957

[B27] Kryger AI Lassen CF Andersen JH The role of physical examination in studies of musculoskeletal disorders of the elbow Occup Environ Med 2007 64 776 781 10.1136/oem.2005.026260 17522132 PMC2078422

[B28] Lewis JS Valentine RE The pectoralis minor length test: A study of the intra-rater reliability and diagnostic accuracy in subjects with and without shoulder symptoms BMC Musculoskelet Disord 2007 8 64 10.1186/1471-2474-8-64 17620136 PMC1934353

[B29] McCarthy CJ Gittins M Roberts C Oldham JA The reliability of the clinical tests and questions recommended in international guidelines for low back pain Spine 2007 32 921 926 10.1097/01.brs.0000259864.21869.26 17426640

[B30] McEwan I Herrington L Thom J The validity of clinical measures of patella position Man Ther 2007 12 226 230 10.1016/j.math.2006.06.013 16963310

[B31] Myers JB Oyama S Wassinger CA Ricci RD Abt JP Reliability, precision, accuracy, and validity of posterior shoulder tightness assessment in overhead athletes Am J Sports Med 2007 35 1922 1930 10.1177/0363546507304142 17609529

[B32] Neumann PB Grimmer-Somers KA Gill VA Grant RE Rater reliability of pelvic floor muscle strength Aust NZ Continence J 2007 13 8 14

[B33] Peeler J Anderson JE Reliability of the Thomas test for assessing range of motion about the hip Phys Ther Sport 2007 8 14 21 10.1016/j.ptsp.2006.09.023

[B34] Rainville J Noto DJ Jouve C Jenis L Assessment of forearm pronation strength in C6 and C7 radiculopathies Spine 2007 32 72 75 10.1097/01.brs.0000251002.39417.2c 17202895

[B35] Robinson HS Brox JI Robinson R Bjelland E Solem S Telje T The reliability of selected motion- and pain provocation tests for the sacroiliac joint Man Ther 2007 12 72 79 10.1016/j.math.2005.09.004 16843031

[B36] Roussel NA Nijs J Truijen S Smeuninx L Stassijns G Low back pain: Clinimetric properties of the trendelenburg test, active straight leg raise test, and breathing pattern during active straight leg raising J Manipulaitve Physiol Ther 2007 30 270 278 10.1016/j.jmpt.2007.03.001 17509436

[B37] Savic G Bergstrom EMK Frankel HL Jamous MA Jones PW Inter-rater reliability of motor and sensory examinations performed according to American Spinal Injury Association standards Spinal Cord 2007 45 444 451 10.1038/sj.sc.3102044 17387316

[B38] Schneider M Homonai R Moreland B Delitto A Interexaminer reliability of the prone leg length analysis procedure J Manipulative Physio Ther 2007 30 514 521 10.1016/j.jmpt.2007.07.001 17870420

[B39] Sedaghat N Latimer J Maher C Wisebey-Roth T The reproducibility of a clinical grading system of motor control in patients with low back pain J Manipulative Physiol Ther 2007 30 501 508 10.1016/j.jmpt.2007.07.008 17870418

[B40] Visscher CM Lobbezoo F Naeije M A reliability study of dynamic and static pain tests in temporomandibular disorder patients J Orofac Pain 2007 21 39 45 17312640

[B41] Fleiss J Statistical methods for rates and proportions 2003 3 Hoboken, N.J.: Wiley-Interscience

[B42] Mokkink LB Terwee CB Gibbons E Stratford PW Alonso J Patrick DL Knol DL Bouter LM de Vet HCW Inter-rater agreement and reliability of the COSMIN (Consensus-based Standards for the selection of health status Measurement Instruments) Checklist BMC Med Res Methodol 2010 10 8 10.1186/1471-2288-10-8 20860789 PMC2957386

